# Aging Stability and Radical Activity of Plasma-Activated Water Treated in Liquid- and Gas-Phase Reactors

**DOI:** 10.3390/molecules30234585

**Published:** 2025-11-28

**Authors:** Ivan Karlo Cingesar, Višnja Stulić, Franka Markić, Senada Muratović, Mia Kurek, Zoran Herceg, Nadica Maltar-Strmečki, Tomislava Vukušić Pavičić

**Affiliations:** 1 Faculty of Food Technology and Biotechnology, University of Zagreb, Pierottijeva 6, 10000 Zagreb, Croatia; ivan.karlo.cingesar@pbf.unizg.hr (I.K.C.); visnja.stulic@pbf.unizg.hr (V.S.);; 2 Ruđer Bošković Institute, Bijenička c. 54, 10000 Zagreb, Croatianstrm@irb.hr (N.M.-S.)

**Keywords:** plasma activated water, reactive species, storage, EPR

## Abstract

Plasma-activated water (PAW) is a liquid enriched with reactive oxygen and nitrogen species (RONS), which impart antimicrobial and bioactive properties. In this study, PAW generated in liquid or gas phase under nitrogen or oxygen atmospheres was characterized in terms of pH, electrical conductivity, oxidation-reduction potential, surface tension, and concentrations of H_2_O_2_ and NO_2_^−^. Hydroxyl radical (•OH) formation was confirmed using DIPPMPO as a spin-trapping probe, while antioxidant activity was determined directly in treated water for the first time. The stability of reactive species was assessed over three months at room temperature, 4 °C, and −18 °C. Results indicate that plasma effects on physicochemical parameters depend strongly on the process gas. From a long-term storage perspective, samples maintained at 4 °C stabilized at higher H_2_O_2_ and NO_2_^−^ concentrations. Antioxidant activity persisted for up to 60 days, though at low levels. EPR analysis revealed that hydroxyl radical concentration increased slightly during storage, with 60-day samples showing higher signal intensities compared to fresh PAW. Overall, the findings provide new insights into PAW composition, radical dynamics, and stability, highlighting the influence of gas atmosphere and storage conditions on its properties and supporting its potential for applications in the food, agriculture, and biomedical sectors.

## 1. Introduction

Plasma is an ionized gas consisting of ions, excited partials, free radicals, and electrons that emits ultraviolet light. It has been gaining increasing attention due to its potential applications in the food industry, agriculture, medicine, and environmental remediation [1,2]. Based on temperature, plasma can be classified as either thermal plasma (TP) or non-thermal plasma (NTP). Non-thermal plasmas (NTPs) produced at atmospheric pressure, such as corona discharges, plasma jets, and dielectric barrier discharges, are of primary scientific interest because of their easy application. Plasma discharges in water lead to the formation of plasma-activated water (PAW). Its environmentally friendly nature, resulting from the absence of harmful chemical residues, makes it an attractive alternative to conventional chemical disinfectants. However, the composition and stability of PAW strongly depend on plasma parameters, treatment time, water composition, and storage conditions, highlighting the need for systematic optimization to tailor PAW for specific applications [3]. Depending on the above-mentioned parameters, plasma-activated water contains reactive oxygen species (ROS) and reactive nitrogen species (RNS), including hydrogen peroxide (H_2_O_2_), nitrate (NO_3_^−^), nitrite (NO_2_^−^), hydroxyl radicals (•OH), and ozone (O_3_) [4,5]. Reactive species concentrations depend on the plasma generation environment, exhibiting different characteristics in liquid-phase compared to gas-phase plasmas. The generated species remain dissolved in water, conferring antimicrobial, antifungal, and antiviral properties [6]. These reactive species influence the physicochemical properties of plasma-activated water (PAW), such as pH, oxidation-reduction potential (ORP), and electrical conductivity, and play a central role in its biological activity. The presence of long-lived species like hydrogen peroxide, nitrite, and nitrate ensures the persistence of antimicrobial effects, while short-lived radicals such as hydroxyl radicals and ozone contribute to rapid oxidative damage. The synergistic action of these reactive species enables PAW to inactivate a broad spectrum of microorganisms through mechanisms including cell membrane disruption, protein oxidation, and DNA damage [6].

Besides EPR—which remains the most direct and sensitive method for detecting radical species in PAW—a range of complementary spectroscopic and colorimetric techniques is available. These include UV–Vis spectroscopy for characteristic absorption bands, fluorescence probes for hydroxyl radicals, and chemiluminescence for reactive nitrogen species. Although indirect, such methods enable rapid and reproducible quantification of long-lived species and provide useful cross-validation to EPR data. Given that PAW composition can vary with plasma source, operating parameters, and storage conditions, reliable identification of both short- and long-lived radicals is crucial for ensuring consistent PAW quality and linking its chemical composition to functional effects in industrial applications.

Electron Paramagnetic Resonance (EPR), also known as Electron Spin Resonance (ESR), is a spectroscopic method used to study substances with unpaired electrons [7,8]. It operates by detecting how these electrons interact with a magnetic field under microwave radiation. Due to its high sensitivity and precision, EPR is a valuable tool for detecting and quantifying of free radicals in chemistry, physics, and biology. It is widely applied to investigate reaction mechanisms, biological systems, and the properties of advanced materials. Plasma-activated water generates a wide range of unpaired radicals. Therefore, EPR spectroscopy is an irreplaceable technique for characterization and the only one that can directly determine the type and quantity of radicals, making it necessary in the production, storage, and application of PAW. Among these, hydroxyl radicals (•OH) play a key role and are primarily responsible for its antimicrobial properties [6,9]. Detection of short-lived free radicals can be challenging and often unsuccessful. Therefore, in EPR measurements, the so-called spin-trapping method is employed, in which free radicals are trapped by diamagnetic species known as spin traps. Spin trapping involves the addition of a spin trap to samples containing free radicals, allowing them to react and form more stable, long-lived spin adducts that are EPR-detectable. Each spin adduct produces a characteristic EPR spectrum specific to the radical being trapped. To detect hydroxyl radicals in PAW, a 5-diisopropoxyphosphoryl-5-methyl-1-pyrroline N-oxide (DIPPMPO) EPR spin trap, which produces more stable spin adducts, was used for the first time in this study to improve control and optimization of PAW storage and to encourage its application. Therefore, in this study, plasma-activated water (PAW) was generated using both liquid-phase and gas-phase plasma reactors, with the injection of different gases (oxygen, and nitrogen). Plasma was applied at voltages of 20 and 30 kV. The physicochemical properties of the resulting PAW were characterized, and their stability was monitored under different storage conditions (room temperature, 4 °C, and −18 °C) over a period of three months.

## 2. Results and Discussion

### 2.1. Sample Lists with Details

Table 1 presents the sample identifiers (IDs) along with the corresponding process parameters, i.e., independent variables: treatment duration (t), type of gas, NTP phase, and storage temperature. Table 2 presents details on the PAW treatment parameters.

The determined energy in kJ of a pulse in treatment (Table 2) shows a statistically significant difference with respect to gas type, NTP phase, and the interaction between gas type × NTP phase (*p* ≤ 0.000). The specific energy is lower in the N_2_ gas NTP phase compared to the N_2_ liquid NTP phase, while in the O_2_ case, the values are reversed. Comparing the values between N_2_ and O_2_ atmospheres, the process conducted using O_2_ shows significantly lower values of total and specific energy.

### 2.2. Surface Tension Measurements

**Figure 1:**
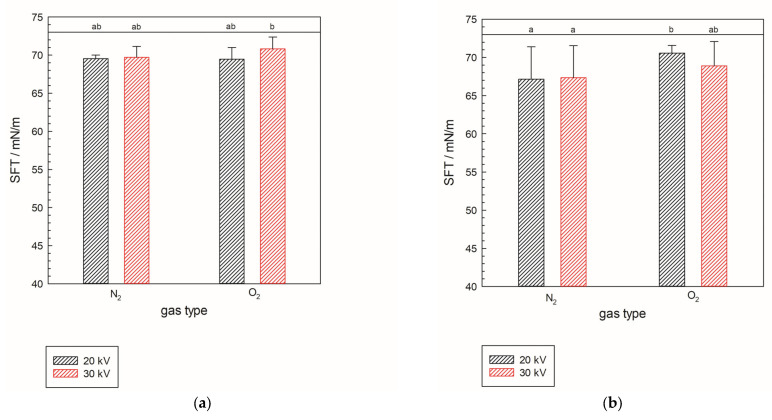
Surface tension of samples treated with gas-phase (**a**) and liquid-phase plasma (**b**) in two different gases, N_2_ and O_2_, at 25 °C. The black line represents a value of 73 mN/m, i.e., the surface tension of pure demineralized water used as reference. Significant differences have been shown with a significance level of *p* ≤ 0.05 within full factorial design (power × gas type × NTP phase).

Surface tension (SFT) generally reflects the influence of plasma treatment on its value. All the analyzed samples show a decrease in SFT value. The largest decrease in value is shown for N_2_-treated samples for treatment parameters of 7 min at 20 kV in the liquid configuration of the NTP reactor, with a value of 67.13 mN/m at 25 °C. Samples treated in liquid-phase plasma for each gas have SFT values lower than their corresponding gas-phase plasma samples. All values (Figure 1a,b) show a significant drop in the SFT value of pure demineralized water, which is around 73 mN/m at 25 °C [10]. The statistical analysis for the three independent variables—power (A), gas type (B), and NTP phase (C)—each with two levels, was conducted to examine their effects on SFT. The *p*-values were evaluated for the three main effects, that is, the individual effect of each factor (A, B, and C), then for the three two-way interactions (A × B, B × C, A × C), and finally the three-way interaction (A × B × C). The analysis shows a significant effect for B (gas type) as one of the main factors (*p* = 0.0356), while power (A) and NTP phase (C) did not show statistical significance, with *p* = 0.5009 and *p* = 0.97995, respectively. Furthermore, the two-way interactions (pA × B = 0.81627, pA × C = 0.28652, pB × C = 0.16441) and the three-way interaction (pA × B × C = 0.2754) do not show statistical significance, indicating that gas type is a critical factor. This confirms that the drop in SFT value results from molecular changes. In other words, plasma-activated water containing ROS and RNS influences changes in surface tension [11]. Furthermore, SFT values show strong negative correlation with the specific energy values (r^2^ = −0.798330) confirming that the gas type is the major factor in SFT change.

It was previously shown (Zhu et al. [12]) that the electric field generated in water during plasma treatment lowers surface tension, allowing the gaseous molecules to dissolve in water easily. Additionally, when, for example, NO_2_^−^ is dissolved in water, it forms electrostatic dipole–dipole interactions with water molecules, thereby lowering the number of formed hydrogen bonds between water molecules, thus lowering the surface tension. The same results were obtained in experiments conducted for all the treated samples in different working gas atmospheres and voltages. The results obtained in this research show the same trends as those observed by Zhu et al. [12].

### 2.3. Reactive Oxygen and Nitrogen Species

The results are presented in Table 3, Table 4, Table 5, Table 6, Table 7, Table 8, Table 9 and Table 10, each showing paired data in respect to gas, N_2_ or O_2_. Samples G1 and G2 were stored at room temperature, G3 and G4 at 4 °C, and G5 and G6 at −18 °C. It is important to note that samples G1, G3, and G5, as well as G2, G4, and G6, underwent identical treatments (20 V or 30 V, respectively), differing only in their storage temperature. As shown in Table 3, Table 4, Table 5, Table 6, Table 7, Table 8, Table 9 and Table 10, statistical analysis for the three independent variables—power (A), treatment time (B), and storage temperature (C)—was conducted, each with two levels, to examine their effects on the dependent variables: concentrations of reactive oxygen and nitrogen species. The *p*-values were evaluated for the three main effects; that is, the individual effect of each factor (A, B, and C), then for the three two-way interactions (A × B, B × C, A × C), and finally for the three-way interaction (A × B × C). In Table 3, Table 4, Table 5, Table 6, Table 7, Table 8, Table 9 and Table 10, uppercase letters within a row indicate values that are not significantly different at a probability of 0.05, while the same lowercase letters within a column indicate values that are not significantly different at a probability of 0.05. This design was used to investigate the effects of storage temperature and duration on reactive oxygen and nitrogen species (ROS and RNS) concentrations. As reported in the literature, higher storage temperatures tend to result in increased generation but more rapid degradation of RONS, whereas lower storage temperatures promote better preservation of the reactive species already present in PAW [13]. Concentration levels of H_2_O_2_ and NO_2_^−^ were monitored for 3 months, because those two types of species are considered more stable compared to hydroxy radicals or ozone [14]. The focus of this research was the period from 14th to 105th day as this presents the real application time used in the food industry, from production to final customer. The concentration of reactive molecular species decreases over time, e.g., H_2_O_2_ or NO_2_^−^, as these species degrade into more stable forms [15].

Comparing nitrogen- (Table 3) and oxygen-treated samples (Table 4) stored at room temperature, samples show similar changes in concentration—an increase in concentration with later decrease. This concentration is affected by both the decomposition of H_2_O_2_, and the recombination of hydroperoxyl radicals to H_2_O_2_. After 3 weeks, both pairs of analyzed samples showed stabilization of concentrations, with N_2_ samples maintaining concentrations around 2 mg/L, and O_2_ samples between 2 and 2.5 mg/L. Both groups of samples showed a slight increase in concentration after the first few days, with a drop in concentration occurring for all samples afterwards. Samples stored at 4 °C (N_2_ G3 and G4, O_2_ G3 and G4) showed a slight peak around day 30. N_2_ samples treated with gas-phase plasma showed a higher peak than O_2_ samples. After 30 days of storage, samples for both gases showed signs of stabilization, with N_2_ samples stabilizing around 3 mg/L, and O_2_ samples around 2.5 mg/L. It is interesting to note that sample O_2_ G3 shows stabilization at a higher H_2_O_2_ concentration than O_2_ G4, even though the O_2_ G4 sample was treated at a higher voltage, usually meaning a higher concentration of H_2_O_2_ [16]. Samples stored at −18 °C remained largely stable, with N_2_-treated samples maintaining concentrations of approximately 3 mg/L, while O_2_-treated samples stabilized slightly later at around 2.5 mg/L. Overall, when comparing the results of H_2_O_2_ concentrations for gas-phase plasma for both nitrogen and oxygen gases, nitrogen shows more fluctuations in concentration over time, while oxygen samples show a more uniform decrease in concentration with smaller peaks through days. From a long-term storage perspective (3 months), samples stored at 4 °C stabilized at higher H_2_O_2_ concentrations, suggesting that they could remain effective for extended use in applications involving contact with surfaces and/or food.

When samples stored at room temperature are compared (N_2_ G1 and G2, O_2_ G1 and G2), samples treated in nitrogen (Table 5) with gas-phase plasma showed higher concentrations of NO_2_^−^. As expected, due to the spontaneous reaction and recombination of NO_2_^−^ [15], the measurements for all the samples showed a decrease in NO_2_^−^ concentrations over time, leading to zero in the case of oxygen-treated samples (O_2_ G1 and G2), and concentrations around 0.03 mg/L for sample N_2_ G5 and 0.01 mg/L for sample N_2_ G6. Samples stored at 4 °C generally exhibited the longest stability of NO_2_^−^ concentrations, maintaining levels above zero, except for the O_2_ G3 (Table 6) sample, which dropped almost immediately to 0 mg/L. It is interesting to note that N_2_ G3 shows a constant drop in concentration but measures higher concentrations than N_2_ G4 after 100 days. Samples kept at −18 °C showed lower concentrations of NO_2_^−^ even after 2 weeks of storage in the freezer, eventually dropping to very low (<0.2 mg/L) concentrations after 4 weeks.

Table 7 shows that N_2_ treatments kept levels comparatively constant, with very slight fluctuations around 2.55 mg/L. Table 8 shows bigger fluctuations in O_2_ samples, stabilizing at slightly lower concentrations except for sample O_2_ L2. According to these findings, nitrogen atmosphere setting favors a more balanced production–scavenging regime from the start, while oxygen atmosphere conditions promote an early increase in H_2_O_2_ concentration, followed by rapid consumption. Increased production of ROS and subsequent enzymatic breakdown have been connected to similar short-term overshoots in H_2_O_2_ in oxygenated environments [17].

A comparable pattern was observed in L3 and L4. Again, O_2_ treatments showed lower concentrations after 14 days but increased to values consistent with N_2_ treatments approximately. L5 and L6 O2 treatments increased to 2.5 to 3.0 mg/L, reaching a similar value to N_2_ samples. At the 3-month mark, both treatments reached nearly identical concentration levels.

The observed differences between N_2_ and O_2_ conditions suggest that oxygen availability plays a central role in regulating H_2_O_2_ dynamics. The faster decline under O_2_ is likely linked to enhanced oxidative processes, including catalase and peroxidase activity, as well as possible Fenton-type reactions that accelerate H_2_O_2_ decomposition [18]. The more stable concentration profiles under O_2_ further indicate a balance between H_2_O_2_ degradation and production, preventing the transient fluctuations observed under N_2_ gas. In contrast, the variability in N_2_ samples, particularly the transient rise in G3 and delayed stabilization in G5–G6, may reflect limited enzymatic activity and slower redox cycling, conditions known to permit temporary accumulation of reactive oxygen species [19]. These findings align with studies showing that oxygen-rich environments favor rapid ROS turnover, while oxygen-limited systems allow higher variability and persistence of H_2_O_2_ [20]. Overall, the results highlight that O_2_ promotes a faster and more controlled reduction in H_2_O_2_, whereas N_2_ maintains a slower and less predictable dynamic.

As shown in Table 9, the NO_2_ concentration in the N_2_ L1 sample was nearly zero, whereas the N_2_L2 sample still retained 0.6 mg/L of NO_2_. The room-temperature oxygen samples (Table 10), O_2_ L1 and N_2_ L2, demonstrate that oxygen liquid-phase plasma does not produce NO_2_^−^ species. The N_2_ L3 and N_2_ L4 samples that were kept at 4 °C behaved similarly to the samples that were kept at room temperature, although the concentration increase peak was considerably larger and it decreased over time like the previous samples. For samples N_2_ L3 and N_2_ L4, the concentration stabilizes at about 0.2 and 0.02 mg/L.

The concentration of the oxygen plasma-treated samples (Table 10) shows nearly 0 mg/L NO_2_^−^ concentration for all the samples. N_2_ promotes quicker NO_2_^−^ breakdown at lower starting concentrations, but O_2_ postpones complete elimination, which is consistent with research indicating O_2_ can promote NO_2_^−^ stabilization [21,22].

In this study, a comparison was made between gas-phase and liquid-phase plasma processes operated under nitrogen and oxygen atmospheres. The results demonstrate that gas-phase plasma generally produces higher concentrations of H_2_O_2_ than liquid-phase plasma, thereby favoring the gas-phase configuration for peroxide generation. From the perspective of nitrite (NO_2_^−^) formation, the gas-phase process also yielded higher values overall.

Determining which gas atmosphere is preferable is not straightforward, as the optimal choice depends on the intended application of plasma-activated water. While both nitrogen and oxygen atmospheres produced comparable concentrations of H_2_O_2_, they differed substantially in terms of NO_2_^−^ accumulation. Specifically, nitrogen-based gas-phase plasma resulted in elevated NO_2_^−^ levels, which may be advantageous when higher nitrite concentrations are desirable. Conversely, oxygen-based gas-phase plasma generated lower, or in some cases negligible, amounts of NO_2_^−^, making it more suitable for applications where minimal nitrite content is required.

### 2.4. pH, Electrical Conductivity, and Oxidation-Reduction Potential

**Figure 2:**
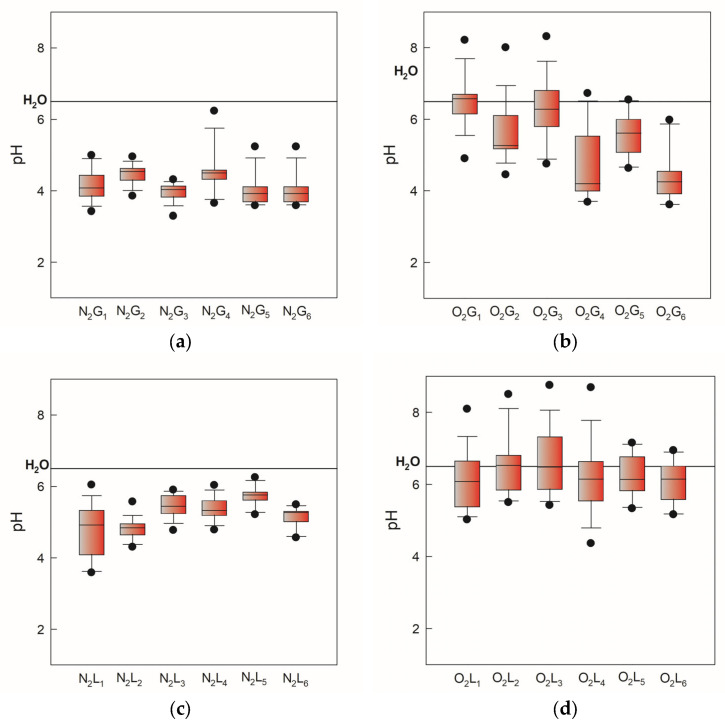
pH values of gas-phase and liquid-phase samples induced by two gases, N_2_ and O_2_, monitored over 105 days, presented as minimum, maximum, and median values. The black line indicates a pH value of 6.5 for demineralized water. (**a**) nitrogen gas-phase samples, (**b**) oxygen gas-phase samples, (**c**) nitrogen liquid-phase samples, (**d**) oxygen liquid-phase samples.

**Figure 3:**
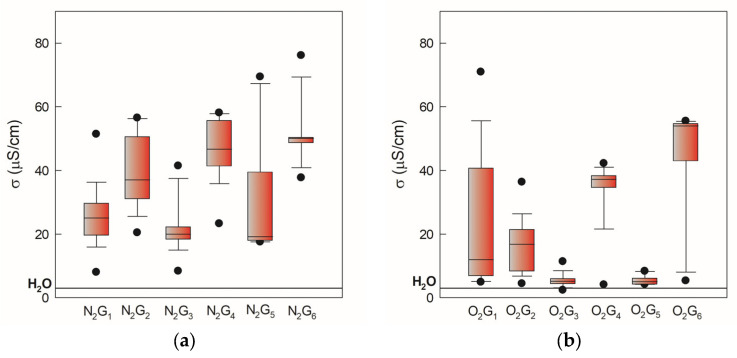

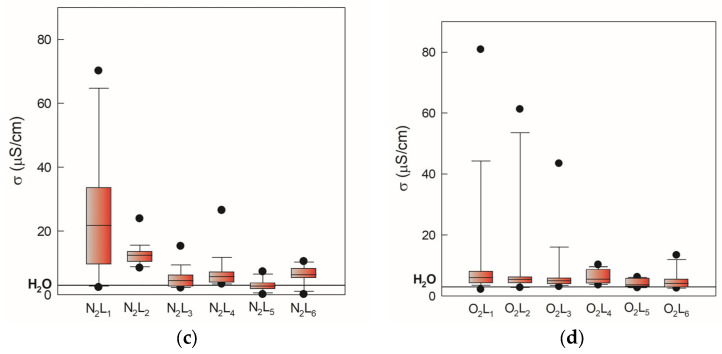
Electrical conductivity of gas-phase and liquid-phase samples induced by two gases, N_2_ and O_2_, monitored over 105 days, presented as minimum, maximum, and median values. The black line indicates electrical conductivity value of 3 mS/cm for demineralized water. (**a**) nitrogen gas-phase samples, (**b**) oxygen gas-phase samples, (**c**) nitrogen liquid-phase samples, (**d**) oxygen liquid-phase samples.

During physicochemical characterization, pH, conductivity, and oxidation-reduction potential (ORP) were measured. In Figure 2, the pH values of gas-phase and liquid-phase samples induced by two gases, N_2_ and O_2_, monitored over 105 days, are presented as minimum, maximum, and median values. Higher values and greater deviation have been observed for oxygen-induced samples in both NTP phases (Figure 2b,d). Electrical conductivity in both atmospheres increased relative to demineralized water (3 mS/cm) as shown in Figure 3. Significant deviation can be attributed to the dependence of electrical conductivity on temperature. This occurs because we followed the storage conditions and measured the conductivity after thawing the PAW, which in real practice changes the measurement temperature by ±5 °C.

The minimum (194.5 mV), maximum (443.6 mV), and median values of ORP obtained in our study (345.5 mV) show an increase compared to the ORP in demineralized water (252.3 mV). In general, an increase is characteristic of the gas phase in both atmospheres, regardless of storage, while a decrease is characteristic of the liquid phase in both atmospheres, also regardless of storage (Table 11). Formation of RONS enables this together with usually lowering pH compared to untreated water [14]. Mechanistically speaking, oxidants generated by plasma (nitrites and/or nitrates) affect ORP increase and drop in pH due to their acidic nature when dissolved [23]. Also, electrical conductivity increases due to the increase in the concentration of dissolved ions [24].

The N_2_ G4 sample (gaseous-phase nitrogen plasma sample stored at 4 °C) has the lowest value of pH, the highest oxidation-reduction potential, and the highest electrical conductivity compared to other samples. The O_2_ L2 sample (liquid-phase oxygen plasma sample stored at room temperature) showed the highest pH, the lowest oxidation-reduction potential, and a mid-range electrical conductivity among all measured values. A similar divergence was observed in pH, which did not consistently decrease under oxygen as reported in the literature. Despite these differences, electrical conductivity in both atmospheres followed the established trend, increasing relative to demineralized water.

The obtained results presented in our study suggest that the effect of plasma on physicochemical parameters is not uniform but depends on the gas used in the process. While both gas- and liquid-phase nitrogen plasma produced results consistent with earlier observations, oxygen plasma appeared to introduce additional dynamics that moderated or even counteracted the typical changes in ORP and pH, depending in which the phase plasma was formed. This discrepancy may reflect differences in the relative balance of oxidizing and reducing species generated under oxygen or nitrogen plasma, or differences in acid–base equilibria related to plasma–liquid interactions [25,26]. As observed in Table 2, Table 4, and Table 8, in plasma-activated water generated using oxygen, H_2_O_2_ concentration increases with higher pH due to greater chemical stability and the formation of the hydroperoxide anion (HO_2_^−^). At lower pH, hydrogen peroxide undergoes faster decomposition through radical-mediated reactions, leading to reduced concentrations. In contrast, as shown in Table 2, Table 3, and Table 7, nitrogen-generated PAW produces fewer reactive oxygen species, limiting H_2_O_2_ formation; however, the reduced presence of highly reactive radicals slows its decomposition, resulting in lower but more stable H_2_O_2_ concentrations at acidic pH. These differences highlight the key role of plasma-generated reactive species and solution pH in the stability of H_2_O_2_ in PAW.

Under the influence of NTP treatment, a chemical imbalance occurs, resulting in the formation of ions, unpaired electrons, and other chemical compounds. The system tends towards a state of minimum energy, leading to the pairing of these species into molecules such as H_2_O_2_ and nitrite as detected in our study (Table 3, Table 4, Table 5, Table 6, Table 7, Table 8, Table 9 and Table 10). This process lasts up to 60 days, during which the concentrations of H_2_O_2_ and nitrite increase, and after 60 days they saturate. After this period, unpaired electrons and compounds containing unpaired electrons remain that can no longer react, and therefore an increase in detected OH− and antioxidant activity is observed as confirmed by EPR spectroscopy.

### 2.5. Antioxidant Activity

To determine the antioxidant activity of PAW samples, the EPR-DPPH method was used, which measures the percentage reduction in the EPR-DPPH signal. From the decrease in signal intensity, the antioxidant properties of the sample can be calculated. For the first time, the antioxidant properties of plasma-activated water were investigated directly. According to the results presented in Table 12, it can be observed that the samples analyzed immediately after the treatment generally showed no antioxidant activity. The only sample that exhibited antioxidant activity, at 20%, was the one treated in gaseous phase with nitrogen gas at a voltage of 30 kV (N_2_G4). In contrast, the samples analyzed 60 days after the treatment showed antioxidant activity, although the values were very low. The table also shows a decrease in antioxidant activity in the case of sample N_2_ G4, which initially exhibited antioxidant activity. The sample that demonstrated the most pronounced antioxidant activity after 60 days of storage was the one treated with oxygen gas at a voltage of 30 kV (O_2_ G4). In addition to sample O_2_ G4, good antioxidant activity was also observed in sample N_2_ G3 (14%).

Since the antioxidant properties were determined directly from the treated water for the first time, it can be assumed that they originate from the balance between oxidizing and reducing species generated during plasma treatment. Due to redox processes induced by plasma-generated species, certain products are formed that contribute to the radical scavenging activity detected in assays such as EPR-DPPH. The results of the antioxidant properties can be correlated with the concentrations of reactive species in PAW, namely hydrogen peroxide and nitrites. Among all samples stored for 60 days, the highest peroxide concentrations were observed in samples N_2_ G3 (2.5 mg/L), N_2_ G4 (2.25 mg/L), and O_2_ G4 (2.55 mg/L), which also exhibited the strongest antioxidant activity measured by EPR. A similar correlation was observed for nitrites, with the highest concentrations after 60 days of storage found in samples N_2_ G3 (0.75 mg/L) and O_2_ G4 (0.58 mg/L). In the literature, studies often focus on the application of plasma-activated water to various products such as fruits and vegetables. These studies have shown that, due to its composition and physicochemical properties, PAW can enhance the antioxidant properties of the treated samples. For instance, Hsu et al. [27] demonstrated that PAW treatment increased the total phenolic and flavonoid contents in water spinach, suggesting an enhancement in antioxidant potential. Similarly, Xiong et al. [28] reported that PAW treatment improved the postharvest quality of shepherd’s purse, including maintaining higher antioxidant activities during storage. Additionally, Zhang et al. [29] found that PAW positively affected color protection and fruit quality, indicating potential improvements in antioxidant properties.

### 2.6. Spin-Trapping Measurements

#### 2.6.1. 0 Days of Storage

**Figure 4:**
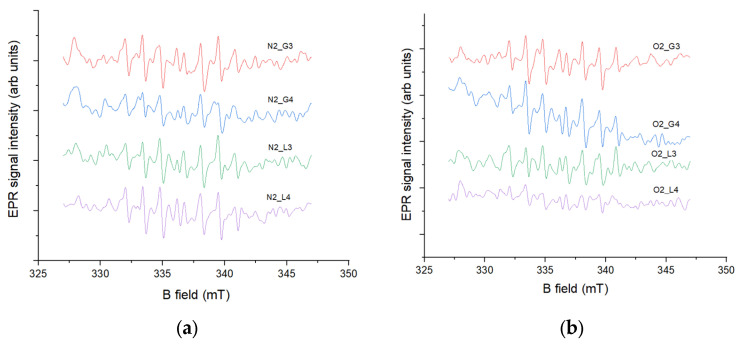
Spin-trapping measurements with DIPPMPO spin trap of (**a**) gas-phase and (**b**) liquid-phase samples induced by two gases conducted directly after HVED treatment of water.

The detection of oxygen radicals, specifically hydroxyl radicals, in this experiment was carried out using an EPR spin-trapping method with the DIPPMPO spin trap. Spin traps are diamagnetic compounds that are highly sensitive to specific radicals; in this case, DIPPMPO is designed to trap hydroxyl radicals. The reaction between the spin trap and the radical forms a paramagnetic compound called a spin adduct, which exhibits a characteristic EPR signal depending on the radical captured. The intensity of this EPR signal is proportional to the concentration of hydroxyl radicals in the sample. According to the results shown in Figure 4, the lowest EPR signal, corresponding to the lowest hydroxyl radical concentration, was observed in samples N_2_ G4 and O_2_ L4, while the highest concentration was observed in samples N_2_ L4 (Figure 4a) and O_2_ G4 (Figure 4b) in the case of oxygen-induced plasma. These results correlate with the antioxidant activity measurements, as the lowest signal was observed in N_2_ G4, the only sample exhibiting antioxidant activity, where the same sample, through its antioxidant effect, reduced the concentration of plasma-generated radicals after treatment. The observed lower EPR signal in oxygen plasma-treated samples can be attributed to the reduced generation of hydroxyl radicals compared to nitrogen plasma. As reported by Takamatsu et al. [30], nitrogen plasma produces significantly higher concentrations of hydroxyl radicals than oxygen plasma under similar experimental conditions, likely due to the distinct chemical dynamics and reactive species present in each plasma. Furthermore, the presence of oxygen in the plasma can alter the chemistry of the treated water, leading to decreased hydroxyl radical production while increasing other reactive species such as H_2_O_2_ and NO_2_, as shown by Uhm et al. [31]. These differences in radical formation help explain the variation in oxidative potential observed between nitrogen- and oxygen-treated samples.

#### 2.6.2. 60 Days of Storage

**Figure 5:**
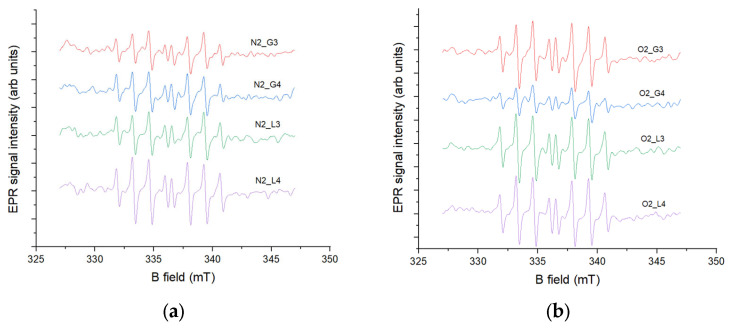
Spin-trapping measurements with DIPPMPO spin trap of (**a**) gas-phase and (**b**) liquid-phase samples induced by two gases conducted 60 days after HVED treatment of water.

Samples stored for 60 days exhibited slightly higher EPR signal intensities compared to the non-stored samples (Figure 5). Among the stored samples, the highest concentrations of hydroxyl radicals were observed in N_2_ L4 (Figure 5a) and O_2_ L4 (Figure 5b), while the lowest were detected in N_2_ G3 and O_2_ G4. These results can also be correlated with the antioxidant activity measurements, showing that samples with lower EPR signal intensities, i.e., lower hydroxyl radical concentrations, exhibited higher antioxidant activity, and opposite. The influence of reactive nitrogen species (RNS) during storage can be explained through their interactions with ROS, particularly hydrogen peroxide, which drive secondary redox processes affecting hydroxyl radical concentrations. Di Meo et al. [32] reported that RNS such as nitrites and nitrates participate in redox cycling, leading to either scavenging or generation of hydroxyl radical depending on the surrounding chemical conditions. Similarly, Chauvin et al. [33] demonstrated that in plasma-treated liquids, the coexistence of ROS and RNS establishes a dynamic equilibrium in which long-lived species (e.g., NO_2_^−^, H_2_O_2_) gradually transform and modulate the levels of short-lived radicals such as hydroxyl. These findings support the observation that stored plasma-activated water exhibits altered hydroxyl radical concentrations and antioxidant activity compared to freshly treated samples.

## 3. Materials and Methods

**Figure 6:**
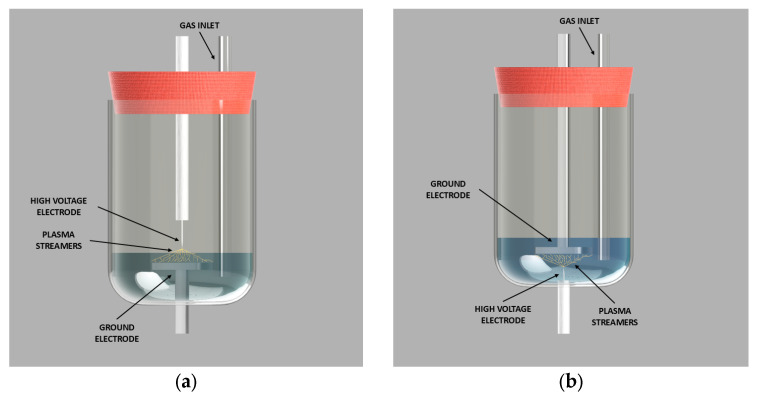
Plasma reactor configuration: (**a**) gaseous phase and (**b**) liquid phase.

Samples of demineralized water (500 mL) were treated using a high-voltage electrical discharge plasma system, developed in collaboration with Impel d.o.o., Zagreb, Croatia. Plasma generation was carried out in either the gaseous (Figure 6a) or liquid (Figure 6b) phase, with nitrogen or oxygen introduced as process gases.

**Figure 7:**
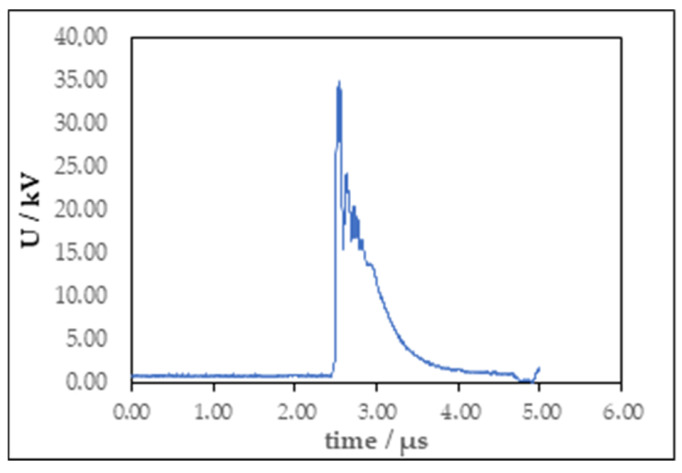
Voltage waveform of gas phase plasma treatment.

All experiments were performed at a frequency of 120 Hz with a pulse duration of 2 µs. The applied voltages were set to 20 or 30 kV and measured using a Tektronix P6015A (Tektronix Inc., Beaverton, OR, USA) high-voltage probe connected to a Tektronix 2 Series mixed-signal oscilloscope for data acquisition (Figure 7). Each treatment lasted 7 min. The diameter of the disc electrode is 45 mm, and the thickness is 5 mm. The needle electrode was 1 mm in diameter and the needle point diameter was 0.1 mm. Distance between electrodes for both reactor configurations was 10 mm.

The plasma was generated in a point-to-plate configuration, where the high-voltage electrode was a titanium needle (point) and the grounded electrode was a stainless steel needle (plate). In gas-phase plasma treatments, the high-voltage electrode was positioned above the liquid while the grounded electrode was submerged. The reactor’s rubber cap was modified to allow both the electrode and the gas inlet tube to pass through. In liquid-phase plasma treatments, both the high-voltage and grounded electrodes, as well as the gas inlet tube, were submerged in the water.

Samples were stored at room temperature, 4 °C, and −18 °C. Samples stored at room temperature and 4 °C were stored in sterile urinary bottles (150 mL maximum volume). Samples stored at −18 °C were frozen in sterile 50 mL falcon tubes filled to 25 mL.

### 3.1. Electrical Conductivity, ORP, and pH

Electrical conductivity was measured using a Hanna Instruments HI76310 probe on a Hanna Instruments (Hanna Instruments Inc., Woonsocket, RI, USA) HI2550 device. Each pH measurement was conducted using a HI1131 probe (Hanna Instruments Inc., Woonsocket, RI, USA), and ORP measurements using a HI3148B probe (Hanna Instruments Inc., Woonsocket, RI, USA) on the same device. Temperature was measured before and after experiments with a contactless IR thermometer (VWR Traceable (Avantor Inc., Radnor Township, PA, USA).

### 3.2. Surface Tension

The surface tension was obtained by the pendant drop method, using a Kruss Drop Shape Analyzer DSA25B goniometer (A.KRÜSS Optronic GmbH, Hamburg, Germany). Measurements were performed at 25 °C by using distilled water as the reference sample and plasma-activated water samples. The drop volume was between 28 and 30 µL. The surface tension was determined after the pendant drop had stabilized. Average values of three drops of the same sample were taken.

### 3.3. Measurement of Hydrogen Peroxide (H_2_O_2_)

Concentration of hydrogen peroxide (mg/L) was determined using UV/VIS spectrometry by adding titan reagent to the sample. Titan reagent was prepared by cooking 1 g of titan dioxide (Thermo Fisher Scientific Inc., Waltham, MA, USA)) in 100 mL concentrated sulphuric acid (Lach-Ner s.r.o., Neratovice, Czech Republic) for 6 h at 190 °C. After cooling overnight, the mixture was transferred to a reagent bottle and diluted to 500 mL with demineralized water. Titan reagent was then stored at 4 °C until use. The spectrophotometric sample was prepared by mixing 2 mL of the PAW sample and 1 mL of prepared titan reagent. Blank sample was prepared by adding 2 mL of distilled water and 1 mL of titan reagent. Samples for analysis were prepared in parallel, and absorbance was measured at 410 nm on a SPECORD 50 plus (Analytik Jena GmbH & Co. KG, Jena, Germany) spectrophotometer. The concentration of hydrogen peroxide (mg/L) was determined using the prepared calibration curve.

### 3.4. Measurement of Nitrite (NO_2_^−^)

The concentration of nitrite ions was determined using UV/VIS spectrometry by adding Griess reagent to the sample. Griess reagent was prepared as follows: solution A: 1% *w*/*v* sulfanilamide in 5% (*v*/*v*) phosphoric acid, and solution B: 0.1% *w*/*v* N-(1-naphthyl) ethylenediamine dihydrochloride (NED) in distilled water. Solutions A and B were then stored in a refrigerator at 4 °C until used. For spectrophotometric analysis, 1 mL of sample was mixed with 1 mL of freshly mixed Griess regent (1:1, A:B). Prepared sample was then incubated for 15 min. Absorbance of samples was measured at 540 nm on SPECORD 50 plus (Analytik Jena, Jena, Germany) spectrophotometer. The concentration of nitrite (mg/L) was determined using the prepared calibration curve. All the samples were analyzed in parallel.

### 3.5. EPR Measurements

All X-band EPR spectra were recorded using a Bruker Magnettech ESR5000 spectrometer (Bruker BioSpin GmbH & Co. KG, Ettlingen, Germany) at room temperature. The antioxidant activity of PAW samples was determined using the 2,2-diphenyl-1-picrylhydrazyl (DPPH) method, in which a decrease in EPR signal amplitude corresponds to the antioxidant activity of the samples. A total of 400 μL of 15 mM DPPH (prepared in 96% ethanol, Gram-mol, Zagreb, Croatia) was mixed with 600 μL of PAW. EPR spectra were recorded over 30 min with the following parameters: magnetic field from 330 to 344 mT, microwave frequency 100 kHz, microwave power 10 mW, and modulation amplitude 0.2 mT. First, a blank sample, containing 400 μL of DPPH and 600 μL of distilled water, was recorded. The antioxidant activity of PAW samples is expressed as a percentage and was calculated using the following formula:

(1) $$\%\;\mathrm{reduction}\;\mathrm{of}\;\mathrm{DPPH}\;=\;\frac{A_{b}-A_{PAW}}{A_{b}}\times 100$$

where the *A*_b_ is the EPR amplitude of the blank sample and *A*_PAW_ is the EPR amplitude of the PAW samples.

Free radicals, generated by PAW, were monitored using the EPR–spin-trapping method. In this case, 5-diisopropoxyphosphory-l-5-methyl-1-pyrroline N-oxide, i.e., DIPPMPO (Focus Biolomecules LLC., Plymouth Meeting, PA, USA) was used. After the treatment, 130 μL distilled water was mixed with 20 μL of 0.35 mM hydrogen peroxide (Gram mol, Zagreb, Croatia), 10 μL of 10 mM DIPPMPO, 130 μL PAW, and 10 μL 0.15 mM iron chloride tetrahydrate (Sigma-Aldrich, Inc., St. Louis, MA, USA). After incubation of 2 min at room temperature, spectra were recorded with the following parameters: magnetic field from 327 mT to 347 mT, microwave frequency 100 kHz, microwave power 10 mT, and modulation amplitude 0.1 mT. The blank sample contained 130 μL of distilled water instead of 130 μL of PAW. All measurements in the experiment were carried out immediately after plasma treatment and after 60 days of storage at 4 °C.

### 3.6. Statistical Analysis

Data collected were expressed as mean standard deviation (SD) and the significance of differences between the control and NTP-treated groups was established using the data analysis software Systat v.13.2.01 (Grafiti LLC, Palo Alto, CA, USA). After testing for normal distribution (Kolmogorov–Smirnov test of normality), the results were analyzed by multivariate analysis of variance (MANOVA) and the differences among the mean values were assessed using Duncan’s multiple range test, where a *p*-value ≤ 0.05 was considered to indicate a significant difference. Details on specific tested interactions have been provided in the manuscript in the paragraphs where the results are presented.

## 4. Conclusions

This study provides a comprehensive evaluation of the physicochemical properties, radical generation, antioxidant activity, and long-term stability of plasma-activated water (PAW) generated under different phases and gas atmospheres. Overall, both total and specific energy increase proportionally with treatment intensity, reflecting the efficiency and stability of the HVED. Gas-phase NTP shows lower energy consumption than liquid-phase NTP, with O_2_ gas-phase NTP being the least energy-demanding process. Statistical analysis proves that the concentrations of H_2_O_2_ and NO_2_^−^ were affected by both storage temperature and storage time. The results demonstrate that the composition and reactivity of PAW are highly dependent on the plasma conditions, with nitrogen and oxygen atmospheres producing distinct effects on pH, conductivity, ORP, surface tension, and concentrations of H_2_O_2_ and NO_2_^−^. Hydroxyl radical formation, confirmed by spin-trapping EPR, and measurable antioxidant activity highlight the reactive potential of PAW. Importantly, storage conditions were found to play a critical role in maintaining stability, with samples preserved at 4 °C retaining higher concentrations of long-lived species such as H_2_O_2_ and NO_2_^−^ compared to room-temperature or frozen storage. Antioxidant activity and radical dynamics persisted, albeit at low levels, up to 60 days. Collectively, these findings advance the understanding of PAW stability and reactivity, emphasizing the significance of plasma generation parameters and storage conditions.

## Figures and Tables

**Table 1:** Sample list with details and IDs, along with the corresponding process parameters.

SAMPLE ID	*U*/kV	*t*/min	Gas	NTP Phase	Storage Temp/°C
N_2_ G1	20	7	nitrogen	gas	25
N_2_ G2	30	7	nitrogen	gas	25
N_2_ G3	20	7	nitrogen	gas	4
N_2_ G4	30	7	nitrogen	gas	4
N_2_ G5	20	7	nitrogen	gas	−18
N_2_ G6	30	7	nitrogen	gas	−18
N_2_ L1	20	7	nitrogen	liquid	25
N_2_ L2	30	7	nitrogen	liquid	25
N_2_ L3	20	7	nitrogen	liquid	4
N_2_ L4	30	7	nitrogen	liquid	4
N_2_ L5	20	7	nitrogen	liquid	−18
N_2_ L6	30	7	nitrogen	liquid	−18
O_2_ G1	20	7	oxygen	gas	25
O_2_ G2	30	7	oxygen	gas	25
O_2_ G3	20	7	oxygen	gas	4
O_2_ G4	30	7	oxygen	gas	4
O_2_ G5	20	7	oxygen	gas	−18
O_2_ G6	30	7	oxygen	gas	−18
O_2_ L1	20	7	oxygen	liquid	25
O_2_ L2	30	7	oxygen	liquid	25
O_2_ L3	20	7	oxygen	liquid	4
O_2_ L4	30	7	oxygen	liquid	4
O_2_ L5	20	7	oxygen	liquid	−18
O_2_ L6	30	7	oxygen	liquid	−18

**Table 2:** Sample list with details of PAW treatment parameters.

SAMPLE ID	*U*/kV	*I*/A	Pulse Energy/kJ	Total Energy/kJ	Specific Energy/kJ/dm^3^
N_2_ G1	20	12.4	0.496	31,248	62,496
N_2_ G2	30	18.6	1.116	70,308	140,616
N_2_ G3	20	12.4	0.496	31,248	62,496
N_2_ G4	30	18.6	1.116	70,308	140,616
N_2_ G5	20	15.4	0.616	31,248	62,496
N_2_ G6	30	27.2	1.632	70,308	140,616
N_2_ L1	20	15.4	0.616	38,808	77,616
N_2_ L2	30	27.2	1.632	102,816	205,632
N_2_ L3	20	2.8	0.112	38,808	77,616
N_2_ L4	30	3.2	0.192	102,816	205,632
N_2_ L5	20	2.8	0.112	38,808	77,616
N_2_ L6	30	3.2	0.192	102,816	205,632
O_2_ G1	20	2.6	0.104	7056	14,112
O_2_ G2	30	2.6	0.156	12,096	24,192
O_2_ G3	20	2.6	0.104	7056	14,112
O_2_ G4	30	2.6	0.156	12,096	24,192
O_2_ G5	20	12.4	0.496	7056	14,112
O_2_ G6	30	18.6	1.116	12,096	24,192
O_2_ L1	20	12.4	0.496	6552	13,104
O_2_ L2	30	18.6	1.116	9828	19,656
O_2_ L3	20	15.4	0.616	6552	13,104
O_2_ L4	30	27.2	1.632	9828	19,656
O_2_ L5	20	15.4	0.616	6552	13,104
O_2_ L6	30	27.2	1.632	9828	19,656

**Table 3:** H_2_O_2_ concentration in nitrogen gas-phase plasma.

H_2_O_2_/mgL^−1^ N_2_ Gas NTP Phase						
20 kV				30 kV		
Days of Storage	T (−18 °C) N_2_ G5	T (4 °C) N_2_ G3	T (20 °C) N_2_ G1	T (−18 °C) N_2_ G6	T (4 °C) N_2_ G4	T (20 °C) N_2_ G2
14	2.98 ± 0.00 ^ABa^	2.68 ± 0.22 ^Aa^	2.33 ± 0.21 ^Ba^	2.63 ± 0.00 ^ABab^	3.12 ± 0.28 ^Ba^	2.21 ± 0.21 ^Aa^
35	2.70 ± 0.20 ^Aa^	3.95 ± 0.28 ^Aa^	2.07 ± 0.03 ^Aa^	2.65 ± 0.20 ^ABab^	3.05 ± 0.15 ^Ba^	2.15 ± 0.13 ^Aa^
56	2.60 ± 0.08 ^Aa^	2.54 ± 0.01 ^Aa^	1.83 ± 0.00 ^Aa^	2.17 ± 0.08 ^Aa^	2.26 ± 0.02 ^Aa^	2.00 ± 0.23 ^Aa^
70	2.68 ± 0.04 ^Aa^	2.90 ± 0.05 ^Aa^	2.13 ± 0.18 ^Aa^	2.44 ± 0.04 ^ABa^	2.77 ± 0.12 ^Ba^	2.16 ± 0.15 ^Aa^
105	3.12 ± 0.18 ^Aa^	3.25 ± 0.03 ^Aa^	2.22 ± 0.08 ^Aa^	2.96 ± 0.18 ^Ab^	3.38 ± 0.67 ^Aa^	2.28 ± 0.16 ^Aa^

**Table 4:** H_2_O_2_ concentration in oxygen gas-phase plasma.

H_2_O_2_/mgL^−1^ O_2_ Gas NTP Phase						
20 kV				30 kV		
Days of Storage	T (−18 °C) O_2_ G5	T (4 °C) O_2_ G3	T (20 °C) O_2_ G1	T (−18 °C) O_2_ G6	T (4 °C) O_2_ G4	T (20 °C) O_2_ G2
14	4.04 ± 3.55 ^Aa^	1.89 ± 0.33 ^Ab^	2.06 ± 0.28 ^Aa^	2.16 ± 0.52 ^Aa^	1.42 ± 0.04 ^Aa^	2.15 ± 0.28 ^Aa^
35	2.51 ± 0.25 ^Aa^	2.87 ± 0.09 ^Aa^	2.50 ± 0.03 ^Aa^	2.87 ± 0.50 ^Aa^	2.36 ± 0.03 ^Ab^	2.50 ± 0.03 ^Aa^
56	2.51 ± 0.49 ^Aa^	2.35 ± 0.15 ^Aab^	2.23 ± 0.16 ^Aa^	2.29 ± 0.09 ^Aa^	2.47 ± 0.15 ^Ab^	2.18 ± 0.16 ^Aa^
70	2.38 ± 0.05 ^Aa^	2.59 ± 0.03 ^Aab^	2.43 ± 0.10 ^Aa^	2.41 ± 0.17 ^Aa^	2.55 ± 0.10 ^Ab^	2.38 ± 0.10 ^Aa^
105	2.36 ± 0.15 ^Aa^	2.79 ± 0.24 ^Aa^	2.45 ± 0.23 ^Aa^	2.54 ± 0.16 ^Aa^	2.47 ± 0.07 ^Ab^	2.27 ± 0.23 ^Aa^

**Table 5:** NO_2_^−^ concentration in nitrogen gas-phase plasma.

NO_2_^−^/mgL^−1^ N_2_ Gas NTP Phase						
	20 kV			30 kV		
Days of Storage	T (−18 °C) N_2_ G5	T (4 °C) N_2_ G3	T (20 °C) N_2_ G1	T (−18 °C) N_2_ G6	T (4 °C) N_2_ G4	T (20 °C) N_2_ G2
14	0.07 ± 0.00 ^Aa^	1.16 ± 0.04 ^Ca^	0.83 ± 0.02 ^Bb^	0.00 ± 0.00 ^Aa^	0.785 ± 0.01 ^Cd^	0.105 ± 0.01 ^Bb^
35	0.00 ± 0.00 ^Aa^	0.79 ± 0.00 ^Aa^	0.58 ± 0.00 ^Aab^	0.00 ± 0.00 ^Aa^	0.23 ± 0.00 ^Ac^	0.00 ± 0.00 ^Aa^
56	0.00 ± 0.00 ^Aa^	0.74 ± 0.01 ^Ba^	0.08 ± 0.02 ^Aa^	0.00 ± 0.00 ^Aa^	0.13 ± 0.01 ^Bb^	0.01 ± 0.01 ^Aa^
70	0.00 ± 0.00 ^Aa^	0.62 ± 0.02 ^Ba^	0.04 ± 0.01 ^Aa^	0.00 ± 0.00 ^Aa^	0.11 ± 0.02 ^Bb^	0.01 ± 0.01 ^Aa^
105	0.00 ± 0.00 ^Aa^	0.45 ± 0.01 ^Ba^	0.00 ± 0.00 ^Aab^	0.00 ± 0.00 ^Aa^	0.02 ± 0.02 ^Aa^	0.18 ± 0.21 ^Ac^

**Table 6:** NO_2_^−^ concentration in oxygen gas-phase plasma.

NO_2_^−^/mgL^−1^ O_2_ Gas NTP Phase						
20 kV				30 kV		
Days of Storage	T (−18 °C) O_2_ G5	T (4 °C) O_2_ G3	T (20 °C) O_2_ G1	T (−18 °C) O_2_ G6	T (4 °C) O_2_ G4	T (20 °C) O_2_ G2
14	0.15 ± 0.00 ^Ad^	0.00 ± 0.00 ^Aa^	0.00 ± 0.00 ^Aa^	0.34 ± 0.00 ^Ab^	1.90 ± 0.00 ^Ae^	0.20 ± 0.00 ^Ac^
35	0.11 ± 0.00 ^Cc^	0.02 ± 0.02 ^Bb^	0.00 ± 0.00 ^Aa^	0.07 ± 0.00 ^Aa^	1.29 ± 0.02 ^Cd^	0.11 ± 0.00 ^Bb^
56	0.05 ± 0.00 ^Bb^	0.00 ± 0.00 ^Aa^	0.00 ± 0.00 ^Aa^	0.02 ± 0.01 ^Aa^	0.98 ± 0.00 ^Bc^	0.00 ± 0.00 ^Aa^
70	0.05 ± 0.00 ^Ab^	0.00 ± 0.00 ^Aa^	0.00 ± 0.00 ^Aa^	0.05 ± 0.06 ^Aa^	0.58 ± 0.02 ^Bb^	0.00 ± 0.00 ^Aa^
105	0.01 ± 0.00 ^Ba^	0.00 ± 0.00 ^Aa^	0.00 ± 0.00 ^Aa^	0.04 ± 0.00 ^Ba^	0.09 ± 0.00 ^Ca^	0.00 ± 0.00 ^Aa^

**Table 7:** H_2_O_2_ concentration in nitrogen liquid-phase plasma.

H_2_O_2_/mgL^−1^ N_2_ Liquid NTP Phase						
20 kV				30 kV		
Days of Storage	T (−18 °C) N_2_ L5	T (4 °C) N_2_ L3	T (20 °C) N_2_ L1	T (−18 °C) N_2_ L6	T (4 °C) N_2_ L4	T (20 °C) N_2_ L2
14	2.10 ± 0.00 ^Aa^	2.42 ± 0.09 ^Aa^	2.25 ± 0.23 ^Aa^	2.50 ± 0.00 ^Ca^	2.15 ± 0.01 ^Ba^	2.70 ± 0.35 ^Aa^
35	2.31 ± 0.00 ^Aa^	2.09 ± 0.02 ^Aa^	2.24 ± 0.31 ^Aa^	2.90 ± 0.06 ^Ba^	2.18 ± 0.13 ^Aa^	2.59 ± 0.18 ^ABa^
56	2.06 ± 0.19 ^Aa^	2.31 ± 0.24 ^Aa^	1.91 ± 0.26 ^Aa^	2.33 ± 0.25 ^Aa^	2.58 ± 0.69 ^Aa^	2.06 ± 0.26 ^Aa^
70	2.62 ± 0.24 ^Aa^	2.73 ± 0.43 ^Aa^	2.48 ± 0.08 ^Aa^	2.61 ± 0.28 ^Aa^	3.40 ± 1.32 ^Aa^	2.19 ± 0.08 ^Aa^
105	2.65 ± 0.16 ^Ba^	2.50 ± 0.09 ^ABa^	2.11 ± 0.05 ^Aa^	2.62 ± 0.39 ^Aa^	2.43 ± 0.20 ^Aa^	2.44 ± 0.05 ^Aa^

**Table 8:** H_2_O_2_ concentration in oxygen liquid-phase plasma.

H_2_O_2_/mgL^−1^ O_2_ Liquid NTP-Phase						
20 kV				30 kV		
Days of Storage	T (−18 °C) O_2_ L5	T (4 °C) O_2_ L3	T (20 °C) O_2_ L1	T (−18 °C) O_2_ L6	T (4 °C) O_2_ L4	T (20 °C) O_2_ L2
14	1.52 ± 0.10 ^Aa^	1.67 ± 0.18 ^Aa^	1.69 ± 0.20 ^Aa^	1.38 ± 0.17 ^Aa^	1.65 ± 0.09 ^Aa^	1.76 ± 0.18 ^Aa^
35	2.45 ± 0.06 ^Ab^	2.46 ± 0.12 ^Ab^	2.63 ± 0.03 ^Ab^	2.78 ± 0.24 ^Ab^	2.51 ± 0.22 ^Aab^	2.41 ± 0.08 ^Ab^
56	2.55 ± 0.13 ^Ab^	2.41 ± 0.06 ^Ab^	2.13 ± 0.11 ^Aab^	2.39 ± 0.22 ^Ab^	2.88 ± 0.52 ^Ab^	2.27 ± 0.18 ^Aab^
70	2.56 ± 0.05 ^Ab^	2.65 ± 0.08 ^Ab^	2.39 ± 0.14 ^Ab^	2.54 ± 0.06 ^Ab^	2.50 ± 0.08 ^Aab^	2.65 ± 0.12 ^Ab^
105	2.55 ± 0.21 ^b^	2.42 ± 0.05 ^b^	2.62 ± 0.10 ^b^	2.43 ± 0.02 ^Ab^	2.35 ± 0.17 ^Aab^	2.65 ± 0.20 ^Ab^

**Table 9:** NO_2_^−^ concentration in nitrogen liquid-phase plasma.

NO_2_^−^/mgL^−1^ N_2_ Liquid NTP Phase						
20 kV				30 kV		
Days of Storage	T (−18 °C) N_2_ L5	T (4 °C) N_2_ L3	T (20 °C) N_2_ L1	T (−18 °C) N_2_ L6	T (4 °C) N_2_ L4	T (20 °C) N_2_ L2
14	0.04 ± 0.00 ^Aa^	0.13 ± 0.01 ^Ba^	0.08 ± 0.01 ^ABa^	0.09 ± 0.00 ^Aa^	0.78 ± 0.02 ^Ba^	0.88 ± 0.01 ^Cc^
35	0.00 ± 0.00 ^Aa^	0.16 ± 0.00 ^Aa^	0.07 ± 0.00 ^Aa^	0.00 ± 0.00 ^Aa^	0.23 ± 0.00 ^Aa^	0.65 ± 0.00 ^Aa^
56	0.00 ± 0.00 ^Aa^	0.15 ± 0.01 ^Ba^	0.02 ± 0.00 ^Aa^	0.00 ± 0.00 ^Aa^	0.13 ± 0.00 ^Ba^	0.70 ± 0.01 ^Cb^
70	0.00 ± 0.00 ^Aa^	0.21 ± 0.02 ^Ba^	0.04 ± 0.05 ^ABa^	0.00 ± 0.00 ^Aa^	0.11 ± 0.00 ^Ba^	0.68 ± 0.01 ^Cab^
105	0.00 ± 0.00 ^Aa^	0.19 ± 0.03 ^Ba^	0.01 ± 0.01 ^Aa^	0.00 ± 0.00 ^Aa^	0.021 ± 0.02 ^Ba^	0.64 ± 0.01 ^Cab^

**Table 10:** NO_2_^−^ concentration in oxygen liquid-phase plasma.

NO_2_^−^/mgL^−1^ O_2_ Liquid NTP Phase						
20 kV				30 kV		
Days of Storage	T (−18 °C) O_2_ L5	T (4 °C) O_2_ L3	T (20 °C) O_2_ L1	T (−18 °C) O_2_ L6	T (4 °C) O_2_ L4	T (20 °C) O_2_ L2
14	0.12 ± 0.00 ^Ab^	0.00 ± 0.00 ^Aa^	0.00 ± 0.00 ^Aa^	0.05 ± 0.00 ^Ab^	0.00 ± 0.00 ^Aa^	0.00 ± 0.00 ^Aa^
35	0.18 ± 0.00 ^Bc^	0.00 ± 0.00 ^Aa^	0.00 ± 0.00 ^Aa^	0.01 ± 0.00 ^Ba^	0.00 ± 0.00 ^Aa^	0.00 ± 0.00 ^Aa^
56	0.00 ± 0.01 ^Aa^	0.00 ± 0.00 ^Aa^	0.00 ± 0.00 ^Aa^	0.01 ± 0.02 ^Aa^	0.00 ± 0.00 ^Aa^	0.00 ± 0.00 ^Aa^
70	0.00 ± 0.00 ^Aa^	0.00 ± 0.00 ^Aa^	0.00 ± 0.01 ^Aa^	0.06 ± 0.00 ^Aa^	0.00 ± 0.00 ^Aa^	0.00 ± 0.00 ^Aa^
105	0.00 ± 0.00 ^Aa^	0.00 ± 0.00 ^Aa^	0.00 ± 0.02 ^Aa^	0.00 ± 0.00 ^Aa^	0.00 ± 0.00 ^Aa^	0.00 ± 0.00 ^Aa^

**Table 11:** ORP values determined at 25 °C and the ratio relative to ORP in demineralized water.

SAMPLE ID	ORP/mV	ORP (Sample)/ORP (H_2_O)	
H_2_O	252.3	1	
N_2_ G1	379.1	1.5025	↗
N_2_ G2	443.6	1.7582	↗
N_2_ G3	379.1	1.5025	↗
N_2_ G4	443.6	1.7582	↗
N_2_ G5	379.1	1.5025	↗
N_2_ G6	443.6	1.7582	↗
N_2_ L1	202.9	0.8042	↙
N_2_ L2	247.1	0.9793	↙
N_2_ L3	202.9	0.8042	↙
N_2_ L4	247.1	0.979	↙
N_2_ L5	202.9	0.8042	↙
N_2_ L6	247.1	0.979	↙
O_2_ G1	350.1	1.3876	↗
O_2_ G2	345.5	1.3694	↗
O_2_ G3	350.1	1.3876	↗
O_2_ G4	345.5	1.3694	↗
O_2_ G5	350.1	1.3876	↗
O_2_ G6	345.5	1.3694	↗
O_2_ L1	205.2	0.8133	↙
O_2_ L2	194.5	0.7709	↙
O_2_ L3	205.2	0.8133	↙
O_2_ L4	194.5	0.7709	↙
O_2_ L5	205.2	0.8133	↙
O_2_ L6	194.5	0.7709	↙

↗ and ↙ represent increase and decrease respectively in value of ORP (Sample)/ORP (H_2_O).

**Table 12:** Results of the EPR-DPPH assay for the samples immediately after NTP treatment and after 60 days of storage at 4 °C.

SAMPLE ID	% Reduction in DPPH After Treatment	% Reduction in DPPH After 60 Days of Storage
N2 G3	0 ± 0.00	14.00 ± 2.45
N2 G4	20.90 ± 6.12	4.85 ± 1.08
N2 L3	0 ± 0.00	0.31 ± 3.01
N2 L4	0 ± 0.00	1.87 ± 0.56
O2 G3	0 ± 0.00	0.70 ± 1.77
O2 G4	0 ± 0.00	19.38 ± 4.00
O2 L3	0 ± 0.00	2.06 ± 0.61
O2 L4	0 ± 0.00	3.59 ± 0.71

## Data Availability

The data presented in this study are available on request from the corresponding author. (please specify the reason for restriction, e.g., the data are not publicly available due to privacy or ethical restrictions.)
